# Emergency Veno-Arterial Extracorporeal Membrane Oxygenation for Pericardial Decompression Syndrome

**DOI:** 10.1155/2022/5440635

**Published:** 2022-09-22

**Authors:** Mohamed Laimoud, Patricia Machado, Andrea Rossi Zadra, Mary Maghirang, Ali Alenazy

**Affiliations:** ^1^King Faisal Specialist Hospital and Research Center, Riyadh, Saudi Arabia; ^2^Critical Care Medicine Department, Cairo University, Cairo, Egypt

## Abstract

**Background:**

Pericardiocentesis is a therapeutic lifesaving intervention for patients presenting with cardiogenic shock due to pericardial effusion with signs of tamponade. Pericardial decompression syndrome (PDS) is a rare fatal complication that may occur after pericardiocentesis. *Case Presentation*. We report a case of a patient with idiopathic primary pulmonary hypertension who presented with massive pericardial effusion complicated with rapid hemodynamic and respiratory deterioration. Gradual therapeutic pericardiocentesis was done but progressive circulatory collapse occurred. Emergent veno-arterial extracorporeal membrane oxygenation (VA-ECMO) was applied. Echocardiography revealed severe right ventricle failure. Unfortunately, the patient developed acute progressive thrombocytopenia and bilaterally diffuse subarachnoid hemorrhage after 4 days of ECMO support.

**Conclusions:**

Therapeutic pericardiocentesis can be occasionally fatal in cases of significant pulmonary hypertension with massive pericardial effusion when complicated by pericardial decompression syndrome. Acute significant thrombocytopenia may occur with VA-ECMO support resulting in fatal bleeding.

## 1. Background

Idiopathic pulmonary artery hypertension (IPAH) is a rare rapidly progressive disease affecting the pulmonary precapillary vasculature and results in right-sided heart failure and death. The World Health Organization (WHO) classified IPAH as a part of group I pulmonary hypertension [1, 2]. The increased pulmonary artery resistance is related to endothelial dysfunction with vasoconstriction, remodeling, and thrombosis. The endothelial dysfunction results from imbalance between different vasoactive substances that affect intracellular nitric oxide, endothelin, and prostacyclin pathways [3, 4].

Pericardiocentesis is a therapeutic lifesaving intervention for patients presenting with cardiogenic shock due to pericardial effusion with signs of tamponade [5, 6]. Pericardial decompression syndrome (PDS) is a rare fatal complication that may occur after pericardiocentesis. It is defined as paradoxical hemodynamic deterioration after successful pericardiocentesis. It was referred to that condition as paradoxical hemodynamic instability or low cardiac output syndrome [7, 8].

## 2. Case Presentation

**Figure 1:**
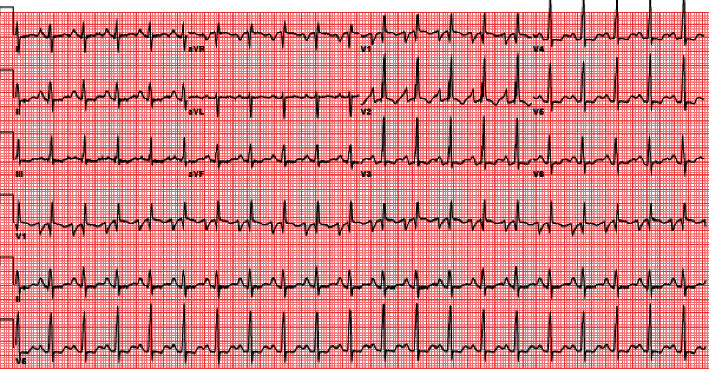
Admission ECG showing sinus tachycardia with right bundle branch block and right axis deviation.

The patient was a 28-year-old female patient with a body mass index of 21.3 (kg/m^2^). She was diagnosed to have idiopathic pulmonary artery hypertension (IPAH) for 3 years and was maintained on maximized pulmonary vasodilators including oral riociguat and macitentan and intravenous continuous infusion of treprostinil. She started to develop progressive right-sided heart failure including bilateral lower limb edema, dyspnea on minimal effort, and pleural and pericardial effusions. She was listed for lung transplantation. She was admitted to our institution with 1 week of exaggerated dyspnea and palpitations. On admission, she presented with sinus tachycardia of 130 beats/min, blood pressure of 90/45 mmHg with presence of jugular venous distention, and pulse oximetry saturation (SPO2%) of 80% on room air. After central venous catheterization, CVP range was 25-30 cm H_2_O (Figure 1).

**Figure 2:**
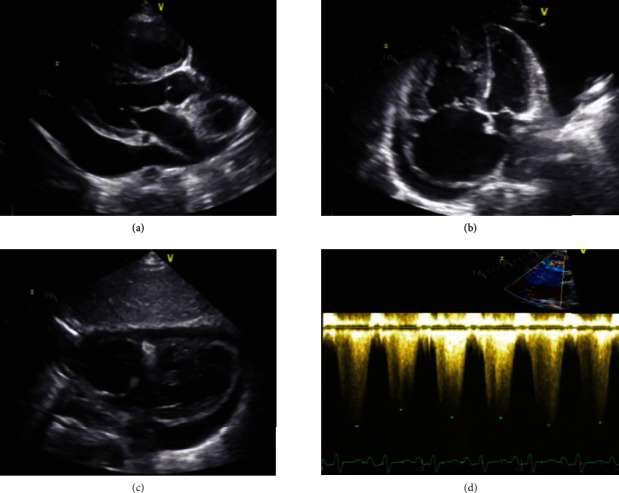
Admission transthoracic echocardiography showing circumferential pericardial effusion, reaching posteriorly 26 mm without compression of right atrium and ventricle. There is normal left ventricular wall thickness and contractility (LVEF > 70%). Severe right ventricular hypertrophy with flattened septum and severe PASP. There is a severe functional TR due to annulus dilatation and tethering of the tricuspid leaflets.

Chest X-ray revealed increased cardiothoracic ratio with increased interstitial thickenings without lung collapse nor consolidation. Laboratory work-up revealed white blood cell count of 6.36 (10^9^/L), platelet count of 189 (10^9^/L), hemoglobin 114 (g/L), serum Na 132 mmol/L, NT-proBNP 3693 pg/mL, normal kidney, and liver chemistries. Septic and virology screening was done including COVID-19 PCR. Echocardiography was done and revealed a large circumferential pericardial effusion without signs of tamponade. Also it showed a severe dilatation of right ventricle with severe systolic dysfunction and flattened interventricular septum. There was a severe right atrium dilatation with left sided shift of interatrial septum. The estimated pulmonary artery systolic pressure (PASP) was more than 115 mmHg while the left ventricle was underfilled but with a good systolic function (Figure 2).

After hemodynamic stabilization with inotropic support and use of high flow nasal oxygen, intravenous frusemide was used to decrease the volume overload as indicated with the generalized anasarca. After debate about the possible benefit and risks of pericardiocentesis, the patient was taken to the catheterization laboratory where pericardiocentesis was done under fluoroscopic and echocardiographic guidance with immediate aspiration of 250 mL pericardial serous fluid. The pericardial effusion was transudate without any pathogen. The analysis revealed the following: fluid protein 38 g/L, albumin 26 g/L, LDH 177 units/L, triglycerides 0.3 mmol/L, no bacteria detected with Gram stain and culture, and no acid fast bacilli detected.

**Figure 3:**
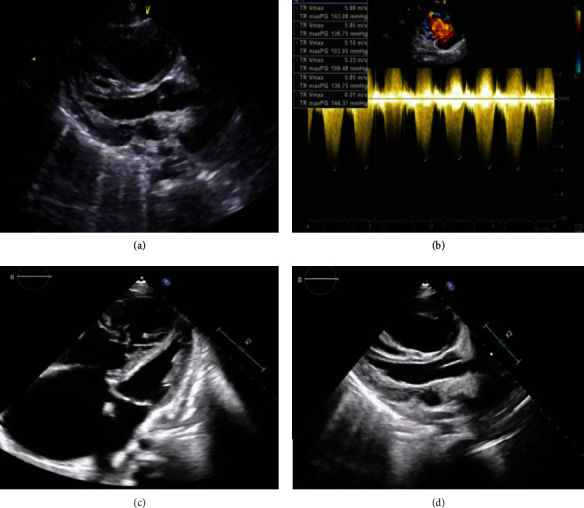
Postpericardiocentesis follow-up transthoracic (a, b) and transesophageal (c, d) echocardiograms showing (1) normal left ventricular dimensions and systolic function, (2) dilated right-sided chambers with reduced RV function, (3) severe functional TR, and (4) no residual pericardial effusion.

Despite gradual withdrawal of 1.3-liter pericardial fluid over the next 24 hours, the patient developed hemodynamic deterioration with progressive lactic acidosis and rising NT-proBNP to 9299 (pg/mL). Emergency echocardiography revealed normal left ventricle while severely dilated right ventricle with systolic dysfunction and increased PASP more than 150 mmHg and minimal pericardial effusion (Figure 3).

Emergent peripheral veno-arterial extracorporeal membrane oxygenation (VA-ECMO) via femoral approach with reperfusion cannula was applied and cardiopulmonary resuscitation was done. Anticoagulation started with unfractionated heparin infusion guided by activated clotting time (ACT) target of 180-220 seconds, heparin level (target 0.3-0.7 units/mL), and antithrombin III activity (target > 50%).The patient had acute kidney injury with decrease of glomerular filtration rate (GFR) from more than 60 to 40 mL/min/1.73 m^2^, but gradual recovery happened without renal replacement therapy. Neurological assessment was frequently done during sedation withdrawal and pupils' size and reactivity to light every 2 hours according to our hospital protocol. Near-infrared spectroscopy (NIRS) was used continuously for monitoring of cerebral and lower limb oxygen saturations. The patient developed a rapid decline of platelet count, and heparin-induced thrombocytopenia (HIT) was unlikely. Despite the HIT 4T score was 6, the heparin–PF4 antibody test was 0.11 (reference ≤ 0.39). Exclusion of significant hemolysis was done by laboratory work-up: blood schistocyte was <0.5%, Coombs test was negative, haptoglobin level was 0.7 (reference: 0.3-2 g/L), peak bilirubin was 42.4 (reference: 0-21 *μ*mol/L), and LDH 585 (reference: 135-214 units/L). The patient developed a thrombus at the tip of ECMO drainage cannula which made the decision to stop anticoagulation exceedingly difficult. So frequent platelet transfusions were done to keep platelet count more than 50 (10^9^/L). Daily sedation withdrawal and neurological assessment was done, and continuous monitoring of brain oxygen saturation by near-infrared spectroscopy (NIRS) was maintained.

**Figure 4:**
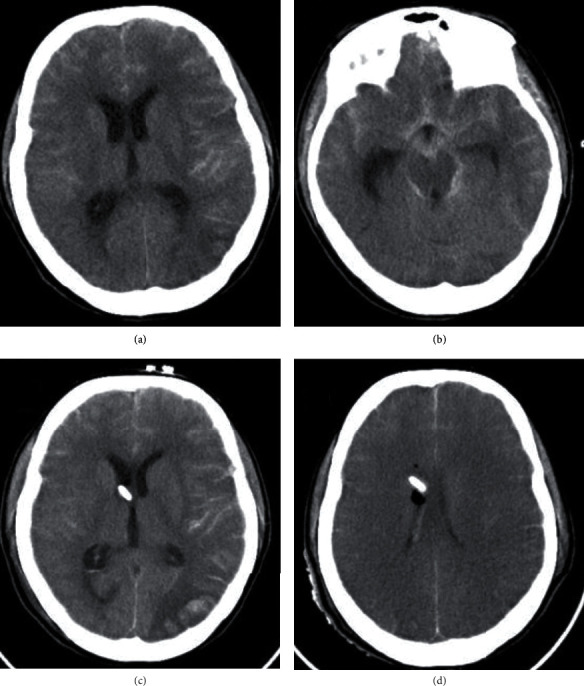
Bilateral diffuse subarachnoid cisterns and intersulcal hyperdensity resembling subarachnoid hemorrhage with a left parieto-occipital cortical and subcortical hypodensity (a, b). Right frontal external ventricular drain with left parietal occipital cortical and subcortical hypodensity with loss of gray-white matter differentiation and focal hyperdensity representing infarction with hemorrhagic transformation (c). Increased cerebral edema with bilateral diffuse loss of gray-white matter differentiation of brain parenchyma (d).

After 4 days of ECMO support, the patient developed accelerated systemic hypertension after sedation withdrawal and deterioration of consciousness; urgent brain computed tomography (CT) imaging revealed left occipital hemorrhagic stroke and bilateral diffuse subarachnoid hemorrhage with intraventricular extension complicated with hydrocephalus. Cerebral angiography excluded aneurysmal dilatation or arteriovenous cerebral malformation. Relief of intracranial hypertension was done using external ventricular draining, head positioning, keeping normocapnia, and osmotic diuresis. Anticoagulation was discontinued immediately when the consciousness was impaired, and platelet count was kept more than 100 (10^9^/L). After 4 days of the cerebral bleeding, the patient developed brain death and brain CT imaging revealed diffuse brain ischemia. Withdrawal of support was done, and the patient was declared dead (Figure 4).

## 3. Discussion

Pericardiocentesis is a therapeutic lifesaving intervention for patients presenting with cardiogenic shock due to pericardial tamponade. It is safe, and the risk of complications ranges from 4% to 10% including coronary artery or cardiac chamber puncture, arrhythmias, hemothorax, and pneumothorax [5, 6]. Pericardial decompression syndrome (PDS) is a rare fatal complication that may occur after pericardiocentesis. It is defined as paradoxical hemodynamic deterioration after successful pericardiocentesis. It was referred to that condition as paradoxical hemodynamic instability or low cardiac output syndrome [7, 8]. Few cases were reported with PDS presented with uni- or biventricular dysfunction [9–14]. Also, few cases were reported with acute pulmonary edema even with normal ventricular function [15–17]. Our patient had already a right ventricular (RV) dysfunction that was aggravated after pericardiocentesis even with slow cautious drainage. After drainage, she had severe right ventricle failure with normal left ventricular function resulting in cardiogenic shock and respiratory failure. Emergent VA-ECMO was applied for rapid resuscitation. Emergency pericardiocentesis is a class IA recommendation for unstable patients and preferably with fluoroscopic and echocardiographic guidance which was done in our patient [5].

Pradhan et al. [18] did analysis of the 35 reported cases of PDS and found the volume of drained fluid ranged from 450 to 2,100 mL, and the onset of hemodynamic deterioration varied from immediate to 48 hours after pericardiocentesis. Most of the presentations were left ventricular failure and pulmonary edema, while RV failure was less frequent presentation. Most of the reported cases of PDS died between 6 hours and 14 days after pericardiocentesis. There are many proposed mechanisms of PDS including rapid drainage of pericardial fluid with rapid increase of venous return and ventricular overloading [9, 10, 17, 19]. In our case, the drainage was slowly done and achieved over 24 hours, but there are no definite guidelines for the proper amount to be drained, especially with preexisting pulmonary hypertension and RV dysfunction. Another proposed mechanism was myocardial stunning and systolic dysfunction due to reduced coronary perfusion pressure [10, 12, 18, 20, 21]. Our echocardiography after deterioration showed normal LV function without regional wall motion abnormalities.

After failure of inotropic support and high flow nasal oxygen to achieve stabilization, rescue VA-ECMO was applied via femoral approach. The use of VA-ECMO is still associated with high mortality and many morbidities including neurological and vascular injuries, bleeding, and thrombocytopenia which were frequently studied [22–29]. The patient developed progressive thrombocytopenia without bleeding, and frequent platelet transfusions were given. Echocardiography was repeated and showed a thrombus at the tip of drainage cannula but did not affect the ECMO flow. Heparin-induced thrombocytopenia (HIT) is a serious problem that can happen after few days of exposure to heparin and associated with arterial and venous thrombotic complications and rarely bleeding [30, 31]. We calculated the 4T score and it showed high probability, but the serology rejected the diagnosis of HIT.

The 4Ts is a scoring for probability of HIT and incorporates 4 items including thrombocytopenia magnitude, timing after exposure to heparin, thrombotic events, and possible other causes of thrombocytopenia. The scores of 0-3, 4-5, and 6-8 were considered as low, intermediate, and high probability for HIT, respectively [32, 33]. Cuker et al. [34] conducted a meta-analysis and concluded that a low probability 4Ts score excludes HIT without need for laboratory testing, while with intermediate or high scores, heparin should be discontinued and laboratory testing should be requested. Moreover, only 7-12% of patients with suspected HIT referred for laboratory testing had positive results [35, 36].

A recent meta-analysis of 12 studies reported the prevalence of thrombocytopenia in 23.2% (95% CI 11.8–34.5; 6 studies) and occurrence of platelet dysfunction during VA-ECMO support without association with ECMO duration [37]. Jiritano et al. [37] reported the decline of platelet count during first 7 days of ECMO initiation and proposed a multifactorial theory including contact with the extracorporeal circuit, inflammatory and coagulative cascade activation, platelet activation, drugs, bleeding in addition to the primary disease, and the hemodynamic deterioration before ECMO initiation. Lukito et al. [38] demonstrated a significantly reduced expression of platelet adhesion receptors with subsequent decreased binding capacity to Von Willebrand factor (vWF) and collagen, leading to platelet dysfunction. Kalbhenn et al. [39] showed a reduced expression of CD62 and CD63, biomarkers of impaired platelet granule secretion with subsequent impaired functional activity of platelets during ECMO support.

The occurrence of intracerebral bleeding was sudden and significant that necessitated neurosurgical intervention to insert external ventricular drain and decrease the intracranial pressure. Female gender, thrombocytopenia, and low body mass index were linked to early intracerebral bleeding in ECMO patients [26, 27, 40].

Finally, our case highlights the importance of gradual judicious decompression during therapeutic pericardiocentesis especially in presence of significant pulmonary hypertension to avoid acute right ventricle failure or pericardial decompression syndrome. Also, careful hemodynamic monitoring after pericardiocentesis should be done for early detection of impaired tissue perfusion and need for cardiopulmonary support. Moreover, acute significant thrombocytopenia may happen shortly after ECMO support and may be complicated with fatal bleeding.

## 4. Conclusions

Therapeutic pericardiocentesis can be occasionally fatal in cases of significant pulmonary hypertension with massive pericardial effusion when complicated by pericardial decompression syndrome. Acute significant thrombocytopenia may occur with VA-ECMO support resulting in fatal bleeding.

## Data Availability

The data of the case study is available from the corresponding author.

## Abbreviations

HIT: Heparin-induced thrombocytopenia
IPAH: Idiopathic pulmonary artery hypertension
LV: Left ventricle
PASP: Pulmonary artery systolic pressure
PDS: Pericardial decompression syndrome
RV: Right ventricle
SPO2: Pulse oximetry saturation
TR: Tricuspid regurgitation
NT-proBNP: N-terminal pro B-type natriuretic peptide
VA-ECMO: Veno-arterial extracorporeal membrane oxygenation
vWF: Von Willebrand factor
NIRS: Near-infrared spectroscopy
WHO: World Health Organization.

## References

[1] Jiao Y. R., Wang W., Lei P. C. (2019). 5-HTT, BMPR2, EDN1, ENG, KCNA5 gene polymorphisms and susceptibility to pulmonary arterial hypertension: a meta-analysis. *Gene*.

[2] McLaughlin V. V., Hoeper M. M., Channick R. N. (2018). Pulmonary arterial hypertension-related morbidity is prognostic for mortality. *Journal of the American College of Cardiology*.

[3] Hemnes A. R., Opotowsky A. R., Assad T. R. (2018). Features associated with discordance between pulmonary arterial wedge pressure and left ventricular end diastolic pressure in clinical practice: implications for pulmonary hypertension classification. *Chest*.

[4] Cao J. Y., Wales K. M., Cordina R., Lau E. M. T., Celermajer D. S. (2018). Pulmonary vasodilator therapies are of no benefit in pulmonary hypertension due to left heart disease: a meta-analysis. *International Journal of Cardiology*.

[5] Adler Y., Charron P., Imazio M. (2015). 2015 ESC guidelines for the diagnosis and management of pericardial diseases: the Task Force for the Diagnosis and Management of Pericardial Diseases of the European Society of Cardiology (ESC) endorsed by: the European Association for Cardiothoracic Surgery (EACTS). *European Heart Journal*.

[6] Chiabrando J. G., Bonaventura A., Vecchié A. (2020). Management of Acute and Recurrent Pericarditis. *Journal of the American College of Cardiology*.

[7] Wagner P. L., McAleer E., Stillwell E. (2011). Pericardial effusions in the cancer population: prognostic factors after pericardial window and the impact of paradoxical hemodynamic instability. *The Journal of Thoracic and Cardiovascular Surgery*.

[8] Dosios T., Stefanidis A., Chatziantoniou C., Sgouropoulou S. (2007). Thorough clinical investigation of low cardiac output syndrome after subxiphoid pericardiostomy. *Angiology*.

[9] Kuroda M., Amano M., Enomoto S. (2001). Severe right ventricular and tricuspid valve dysfunction after pericardiocentesis. *Journal of Medical Ultrasonics*.

[10] Anguera I., Pare C., Perez-Villa F. (1997). Severe right ventricular dysfunction following pericardiocentesis for cardiac tamponade. *International Journal of Cardiology*.

[11] Al Banna R., Husain A. (2011). Reversible severe biventricular dysfunction postpericardiocentesis for tuberculous pericardial tamponade. *BMJ Case Report*.

[12] Ligero C., Leta R., Bayes-Genis A. (2006). Transient biventricular dysfunction following pericardiocentesis. *European Journal of Heart Failure*.

[13] Liao B. T., Lo S. S. (2015). Paradoxical hemodynamic collapse after subxiphoid pericardial window. *A A Case Report*.

[14] Koerner M. M., Alam M., El-Banayosy A. (2015). A case of biventricular failure after pericardial window for large pericardial effusion. *The Heart Surgery Forum*.

[15] Fozing T., Zouri N., Adam O., Oezbek C. (2016). Management of a patient with pericardial decompression syndrome and HOCM. *BMJ Case Report*.

[16] Abdelsalam M., Moritz T. A., Snyder J. A., Cheriyath P., Spizzieri C. L. (2012). Paradoxical hemodynamic instability complicating pericardial window surgery for cardiac tamponade in a cancer patient. *Texas Heart Institute Journal*.

[17] Vandyke W. H., Cure J., Chakko C. S., Gheorghiade M. (1983). Pulmonary edema after pericardiocentesis for cardiac tamponade. *The New England Journal of Medicine*.

[18] Pradhan R., Okabe T., Yoshida K., Angouras D. C., DeCaro M. V., Marhefka G. D. (2015). Patient characteristics, and predictors of mortality associated with pericardial decompression syndrome: a comprehensive analysis of published cases. *European Heart Journal Acute Cardiovascular Care*.

[19] Imazio M. (2015). Pericardial decompression syndrome: a rare but potentially fatal complication of pericardial drainage to be recognized and prevented. *European Heart Journal Acute Cardiovascular Care*.

[20] Sevimli S., Arslan S., Gundogdu F., Senocak H. (2008). Development of left ventricular apical akinesis and thrombus during pericardiocentesis for pericardial tamponade. *Türk Kardiyoloji Derneği Arşivi*.

[21] Wolfe M. W., Edelman E. R. (1993). Transient systolic dysfunction after relief of cardiac tamponade. *Annals of Internal Medicine*.

[22] Schmidt M., Burrell A., Roberts L. (2015). Predicting survival after ECMO for refractory cardiogenic shock: the survival after veno-arterial-ECMO (SAVE)-score. *European Heart Journal*.

[23] Laimoud M., Alanazi M. (2020). The validity of SOFA score to predict mortality in adult patients with cardiogenic shock on venoarterial extracorporeal membrane oxygenation. *Critical Care Research and Practice*.

[24] Rao P., Khalpey Z., Smith R., Burkhoff D., Kociol R. D. (2018). Venoarterial extracorporeal membrane oxygenation for cardiogenic shock and cardiac arrest. *Circulation Heart Failure*.

[25] Chung S., Sheu J., Lin Y. (2012). Outcome of patients with profound cardiogenic shock after cardiopulmonary resuscitation and prompt extracorporeal membrane oxygenation support. *Circulation Journal*.

[26] Laimoud M., Ahmed W. (2020). Acute neurological complications in adult patients with cardiogenic shock on veno-arterial extracorporeal membrane oxygenation support. *The Egyptian Heart Journal*.

[27] Lorusso R., Barili F., Mauro M. D. (2016). In-hospital neurologic complications in adult patients undergoing venoarterial extracorporeal membrane oxygenation: results from the extracorporeal life support organization registry. *Critical Care Medicine*.

[28] Laimoud M., Saad E., Koussayer S. (2021). Acute vascular complications of femoral veno-arterial ECMO: a single-centre retrospective study. *The Egyptian Heart Journal*.

[29] Tanaka D., Hirose H., Cavarocchi N., Entwistle J. W. C. (2016). The impact of vascular complications on survival of patients on venoarterial extracorporeal membrane oxygenation. *The Annals of Thoracic Surgery*.

[30] Ahmed I., Majeed A., Powell R. (2007). Heparin induced thrombocytopenia: diagnosis and management update. *Postgraduate Medical Journal*.

[31] Jang I.-K., Hursting M. J. (2005). When heparins promote thrombosis. *Circulation*.

[32] Lo G. K., Juhl D., Warkentin T. E., Sigouin C. S., Eichler P., Greinacher A. (2006). Evaluation of pretest clinical score (4 T’) for the diagnosis of heparin-induced thrombocytopenia in two clinical settings. *Journal of Thrombosis and Haemostasis*.

[33] Warkentin T. E., Heddle N. M. (2003). Laboratory diagnosis of immune heparin-induced thrombocytopenia. *Current Hematology Reports*.

[34] Cuker A., Gimotty P. A., Crowther M. A., Warkentin T. E. (2012). Predictive value of the 4Ts scoring system for heparin-induced thrombocytopenia: a systematic review and meta-analysis. *Blood*.

[35] Juhl D., Eichler P., Lubenow N., Strobel U., Wessel A., Greinacher A. (2006). Incidence and clinical significance of anti-PF4/heparin antibodies of the IgG, IgM, and IgA class in 755 consecutive patient samples referred for diagnostic testing for heparin-induced thrombocytopenia. *European Journal of Haematology*.

[36] Schallmoser K., Drexler C., Rohde E. (2009). The particle gel immunoassay as a rapid test to rule out heparin-induced thrombocytopenia?. *The Journal of Thoracic and Cardiovascular Surgery*.

[37] Jiritano F., Serraino G. F., Ten Cate H. (2020). Platelets and extra-corporeal membrane oxygenation in adult patients: a systematic review and meta-analysis. *Intensive Care Medicine*.

[38] Lukito P., Wong A., Jing J. (2016). Mechanical circulatory support is associated with loss of platelet receptors glycoprotein Ib*α* and glycoprotein VI. *Journal of Thrombosis and Haemostasis*.

[39] Kalbhenn J., Schlagenhauf A., Rosenfelder S., Schmutz A., Zieger B. (2018). Acquired von Willebrand syndrome and impaired platelet function during venovenous extracorporeal membrane oxygenation: rapid onset and fast recovery. *The Journal of Heart and Lung Transplantation*.

[40] Le Guennec L., Cholet C., Huang F. (2018). Ischemic and hemorrhagic brain injury during venoarterial-extracorporeal membrane oxygenation. *Annals of Intensive Care*.

